# 1-Methyl-D-tryptophan activates aryl hydrocarbon receptor, a pathway associated with bladder cancer progression

**DOI:** 10.1186/s12885-020-07371-6

**Published:** 2020-09-09

**Authors:** Luiz Henrique Gomes Matheus, Stephanie Vanin Dalmazzo, Rodrigo Barbosa Oliveira Brito, Lucas Alves Pereira, Robson José de Almeida, Cleber Pinto Camacho, Humberto Dellê

**Affiliations:** Molecular Innovation and Biotechnology Laboratory, Program in Medicine, Universidade Nove de Julho – UNINOVE, Rua Vergueiro, 235, 2° subsolo, Sao Paulo, CEP: 01504-001 Brazil

**Keywords:** Aryl hydrocarbon receptor, Indoleamine 2, 3-dioxygenase, Bladder cancer, Cytochrome P450 enzymes

## Abstract

**Background:**

Indoleamine 2, 3-dioxygenase-1 (IDO1) is a promising target for immunotherapy in bladder cancer (BC). IDO1 breaks-down tryptophan to generate kynurenine derivatives, which may activate the aryl hydrocarbon receptor (AHR). AHR is an important target for carcinogens, but its association with BC progression was unknown. Two IDO1 inhibitors used in clinical trials are 1-methyl-D-tryptophan (MT) and INCB240360. Because MT is an aromatic hydrocarbon, it may be a ligand for AHR. We hypothesized that AHR could be associated with BC progression and that MT could activate AHR in BC.

**Methods:**

BC patients (*n* = 165) were selected from the Gene Expression Omnibus database. A cut-off point for relative expression of AHR and cytochrome 450 enzymes (CYP1A1, CYP1A2, and CYP1B1; markers of AHR activation) was determined to compare with the grade, stage, and tumor progression. For in vitro experiments, RT4 (grade 1) and T24 (grade 3) BC cells were incubated with MT and INCB240360 to evaluate the expression of AHR and CYP1A1.

**Results:**

AHR activation was associated with grade, stage, and progression of BC. T24 cells express more CYP1A1 than RT4 cells. Although IDO1 expression and kynurenine production are elevated in T24 cells concomitantly to CYP1A1 expression, IDO1 inhibitors were not able to decrease CYP1A1 expression, in contrast, MT significantly increased it in both cell lines.

**Conclusion:**

In conclusion, it is rational to inhibit IDO1 in BC, among other factors because it contributes to AHR activation. However, MT needs to be carefully evaluated for BC because it is an AHR pathway agonist independently of its effects on IDO1.

## Background

Bladder cancer (BC) is the most common malignancy of the urinary tract [1]. Although the non-muscle-invasive is the most common form of BC, a significant part of these cases progress to muscle-invasive form after resection and adjuvant therapies.

The aryl hydrocarbon receptor (AHR) is a conserved transcription factor that responds to several chemicals to mediate the expression of genes that control detoxification, proliferation, transformation, and regulation of the immune system. Ligand-activated AHR translocates into the nucleus to dimerize with ARNT (aryl hydrocarbon receptor nuclear translocator) to modulate the expression of genes that encode enzymes responsible by degradation of its ligand. This process is essential for cellular detoxification, in which cytochrome P450s have direct participation, especially CYP1A1, CYP1A2, and CYP1B1 [2]. The direct effect of the AHR on the gene transcription gives it powerful influence on the carcinogenesis and tumor progression. Therefore, expression of cytochrome P450s is associated with prognosis of melanoma [3]. In addition, the presence of high levels of AHR correlated to poor overall survival in breast cancer patients [4] and its activation promotes invasion of clear cell renal cell carcinoma [5]. Correlating with BC, studies have demonstrated that AHR is involved in the carcinogenesis of the BC since it is activated by tryptophan catabolites to induce the expression of CYP1A1 and other cytochromes, which produce oxidative compounds that promote chromatin and nuclear disruption [6].

Indoleamine 2, 3-dioxigenase 1 (IDO1) is the first and rate-limiting enzyme in the degradation of tryptophan, generating kynurenine and its catabolites. These catabolites have immunomodulatory roles [7], including the protection of the embryos against the maternal immune system during pregnancy [8]. Because IDO1 is overexpressed in several types of cancer, it has been strongly linked to cancer immunoediting [9]. In this context, IDO1 inhibitors are promising in the new generation of immunotherapy for cancer. More than fifty clinical trials have been registered in ClinicalTrials.org using IDO1 inhibitors for cancer treatment (ClinicalTrials.org). Among the IDO1 inhibitors, 1-methyl-D-tryptophan (MT, Indoximod) is a promising agent for anti-cancer therapy, therefore, there are clinical trials using MT [10]. However, MT is able to activate AHR in mesenchymal stromal cells, independently of the IDO1 expression [11].

Seeing that IDO1 inhibitors are promising for BC treatment and that MT can activate AHR, a transcription factor involved in the progression of some types of cancer, we have raised the possibility that MT activates AHR in BC cells, pointing it as inadequate to treat this type of cancer. The primary aim of this study was to verify if AHR activation is associated with BC progression. In addition, to analyze the effect of IDO1 inhibitors on AHR activation in BC cells.

## Methods

### Expression analysis in GEO database

Gene Expression Omnibus (GEO) datasets (http://www.ncbi.nlm.nih.gov/geo/) were explored to obtain data from BC patients [12]. Besides microarray, data of tumor grade, stage and progression were needed. The GSE13507 series was selected, supplying data of 165 patients with transitional-cell carcinoma. All information about patients and their specimens was collected from the studies that originated the GSE13507 series, Lee and coworkers [13], and Kim and coworkers [14]. Tumors were staged and graded according to the American Joint Committee on Cancer. Of all 165, 103 were histologically diagnosed with non-muscle-invasive BC (Ta, *n* = 23; T1, *n* = 80) and 62 with muscle-invasive BC (T2, *n* = 32; T3, *n* = 19; T4, *n* = 11). Nodal/metastatic disease was detected in 13 muscle-invasive cases (T2, *n* = 5; T3, *n* = 5; T4, *n* = 3). Specimens were obtained from transurethral resection of bladder tumor (for superficial tumors) or from cystectomy (for muscle-invasive tumors). Progression was defined as TNM stage progression after disease relapse in non-muscle-invasive tumors and muscle-invasive tumors. GEO2R, an R-based web tool used to analyze GEO data, was used to obtain values of relative expression of AHR, CYP1A1, CYP1A2, and CYP1B1. To separate the patients between the two groups (*low* and *high* expression of the target genes), a cut-off point was determined for each gene based on the median or ROC curve predicting tumor progression. Correlation analysis was performed confronting the relative expression of the target genes with clinicopathological features (gender, age, tumor grade, stage, and progression).

### Cell culture

Human bladder cancer T24 cells and RT4 cells (HTB-4 and HTB-2, respectively; American Type Culture Collection-ATCC, Manassas, VA, USA) were acquired from the Cell Bank of the Federal University of Rio de Janeiro. T24 cells were cultured in RPMI 1640 Medium (Vitrocell, Campinas, Brazil) supplemented with 10% fetal bovine serum (FBS) and penicillin-streptomycin (Sigma-Aldrich, St. Louis, MO) and maintained at 37 °C with 5% CO_2_.

To analyze the effect of IDO inhibitors on the expression of AHR and CYP1A1, RT4 and T24 cells were incubated with 1 μM INCB024360 (INCB, Tocris Bioscience, Bristol, UK) or 1 mM 1-methyl-D-tryptophan (MT, Sigma-Aldrich, St. Louis, MO) for 48 h. INCB was dissolved in DMSO (Sigma-Aldrich, St. Louis, MO) for a 1 mM stock solution. MT was dissolved in 0.1 N NaOH for a 20 mM stock solution and then neutralized to pH 7.4 with 0.1 N HCl.

Cells were incubated into 6-well plates until 80% confluence. At this time, the medium was removed and medium containing IDO inhibitors was added. After 48 h, the cells were harvested and pelleted, being frozen at nitrogen and kept at − 80 °C for storage until RNA extraction. Supernatant was collected and maintained at − 80 °C for kynurenine measurement. The experiments were carried out in triplicates and repeated three times at different times.

### Kynurenine measurement

HPLC was performed to measure kynurenine in the cell culture supernatants. Deproteinization of the samples was performed by centrifugation at 5000 g (15 min at 4 °C) with 10% trichloroacetic acid (1:1, v/v). After centrifugation, the supernatants and the standards were filtered through a 0.22 μm syringe-loaded filter and resolved with a mobile phase of acetonitrile plus sodium acetate buffer (4:96, v/v), pH 4.7. A precolumn of 12.5X4.6 mm and a C18 column (695970–902, Poroshell 120, EC-C18, 4.6x100mm, 4um, Agilent Technologies, Santa Clara, CA, USA) were used. The peak representing kynurenine was detected with the 1220 Infinity II LC Gradient System (G4288B, Agilent Technologies, Santa Clara, USA). A standard curve was built to determine the concentration of the samples (0.5 μM, 1.0 μM, 2.0 μM, 4.0 μM, 8.0 μM, and 16.0 μM).

#### Real-time PCR

Total RNA was extracted from cultured cells using the PureLink® RNA mini kit (12183018A, Invitrogen, California, USA) and PureLink® RNA kit (12,183,016, Invitrogen, California, USA). For cDNA synthesis, SuperScript III Platinum SYBR Green One-Step qRT-PCR Kit (Invitrogen, California, USA) was used. SYBR Green kit (Invitrogen, California, USA Biosystems, California USA) was used in combination with specific primers for IDO1 (sense 5’GGTCATGGAGATGTCCGTAA3’ and antisense 5’ACCAATAGAGAGACCAGGAAGAA3’; NM_002164.5), AHR (sense 5′ ACATCACCTACGCCAGTCGC3’ and antisense 5′ TCTATGCCGCTTGGAAGGAT 3′; NM_001621.4), CYP1A1 (sense 5′ CTATCTGGGCTGTGGGCAA 3′ antisense 5′ CTGGCTCAAGCACAACTTGG 3′; NM_000499.4), and Tata box protein (TBP) as housekeeping (sense 5′ TTCGGAGAGTTCTGGGATTGTA3’ and antisense 5’TTCGGAGAGTTCTGGGATTGTA 3′; NM_003194.4). Triplicate of each sample was heated at 95 °C for 5 min for denaturation, followed by 40 cycles of denaturation at 95 °C for 15 s, annealing at 60 °C for 60 s and extension at 60 °C for 60 s. Reactions were performed in the Applied Biosystems 7500 Real-Time PCR System (Applied Biosystems, Ca, USA). Cycle threshold (Ct) was determined for the housekeeping gene (TBP) as well as target genes using the auto baseline and auto threshold conditions. Normalized gene expression data were made using ΔΔCt (ΔCt reference- ΔCt target) and the 2-ΔΔCt formula.

### Statistical analysis

The data of the GEO datasets were presented as median with the maximum and minimum values or mean and standard deviation. Kolmogorov-Smirnov test was used to verify sample distribution. The analysis of nominal or categorized variables was performed using the chi-square test. To categorize a continuous or ordinal variable, we used the best cut-off point established using a ROC curve and the Youden’s index [15]. For the in vitro data, test-T or ANOVA with post hoc Tukey method was used for analysis between groups. A *p* < 0,05 was considered significant. IBM SPSS Statistics for Windows (Version 22.0 from IBM Corp. Armonk, NY, USA) was used for statistical analysis and GraphPad Prism (version 6; GraphPad Software, Inc.) for graphing.

## Results

### Expression of AHR and cytochromes in BC patients

GSE13507 dataset covered gene expression data from tumor biopsies of 165 patients with NMI and MI BC. The population was separated in *low* and *high* expression of AHR, CYP1A1, CYP1A2, and CYP1B1. According to Table 1, no correlation was observed between AHR expression and the parameters related to tumor progression. However, high expression of CYP1A1 and CYP1A2 was associated with histological grade, tumor stage, and progression. CYP1B1 expression was associated with tumor stage and progression, but not with the histological grade (*p* = 0.375).

**Table 1:** The association between the expression of AHR and cytochromes and the clinicopathological features in BC patients. Data were extracted from the GSE13507 dataset

Parameters	n	AHR		***P***	CYP1A1		***P***	CYP1A2		***P***	CYP1B1		***P***
		Low (%)	High (%)		Low (%)	High (%)		Low (%)	High (%)		Low (%)	High (%)	
**Gender**													
**Female**	30	05 (16.7)	25 (83.3)	*0.149*	26 (86.7)	4 (13.3)	*0.079*	27 (90.0)	03 (10.0)	*0.525*	21 (70.0)	09 (30.0)	*0.060*
**Male**	135	40 (29.6)	95 (70.4)		96 (71.1)	39 (28.9)		126 (93.3)	09 (6.7)		69 (51.1)	66 (48.9)	
**Age (years)**													
**≤65**	69	18 (26.1)	51 (73.9)	*0.772*	50 (72.5)	19 (27.5)	*0.714*	66 (95.7)	03 (4.3)	*0.220*	30 (43.5)	39 (56.5)	0.016*
**> 65**	96	27 (28.1)	69 (71.9)		72 (75.0)	24 (27.5)		87 (90.6)	09 (9.4)		60 (62.5)	36 (37.5)	
**Hist. Grade**													
**Low**	105	31 (29.5)	74 (70.5)	*0.390*	83 (79.0)	22 (21.0)	0.048*	101 (96.2)	04 (3.8)	0.023*	60 (57.1)	45 (42.9)	*0.375*
**High**	60	14 (23.3)	46 (76.7)		39 (65.0)	21 (35.0)		52 (86.7)	08 (13.3)		30 (50.0)	30 (50.0)	
**Stage**													
**Ta-T1**	104	32 (30.8)	72 (69.2)	*0.188*	94 (90.4)	10 (9.6)	0.005*	100 (96.2)	04 (3.8)	0.027*	65 (62.5)	39 (37.5)	0.007*
**>T2**	61	13 (21.3)	48 (78.7)		45 (73.8)	16 (26.2)		53 (86.9)	08 (13.1)		25 (41.0)	36 (59.0)	
**Progression**													
**No**	134	40 (29.9)	94 (70.1)	*0.122*	104 (77.6)	30 (22.4)	0.025*	127 (94.8)	07 (5.2)	0.035*	81 (60.4)	53 (39.6)	0.002*
**Yes**	31	05 (16.1)	26 (83.9)		18 (58.1)	13 (41.9)		26 (83.9)	05 (16.1)		09 (29.0)	22 (71.0)	

All data were analyzed by X^2^ test. Low and High groups were defined using a cutoff point (ROC curve) for expression of AHR, CYP1A1, CYP1A2, and CYP1B1, independently

*Hist. Grade* histological grade, *n* number of BC patients divided by parameters

*p < 0.05

### Expression of IDO1, AHR and CYP1A1 in BC cell lines

**Fig. 1:**
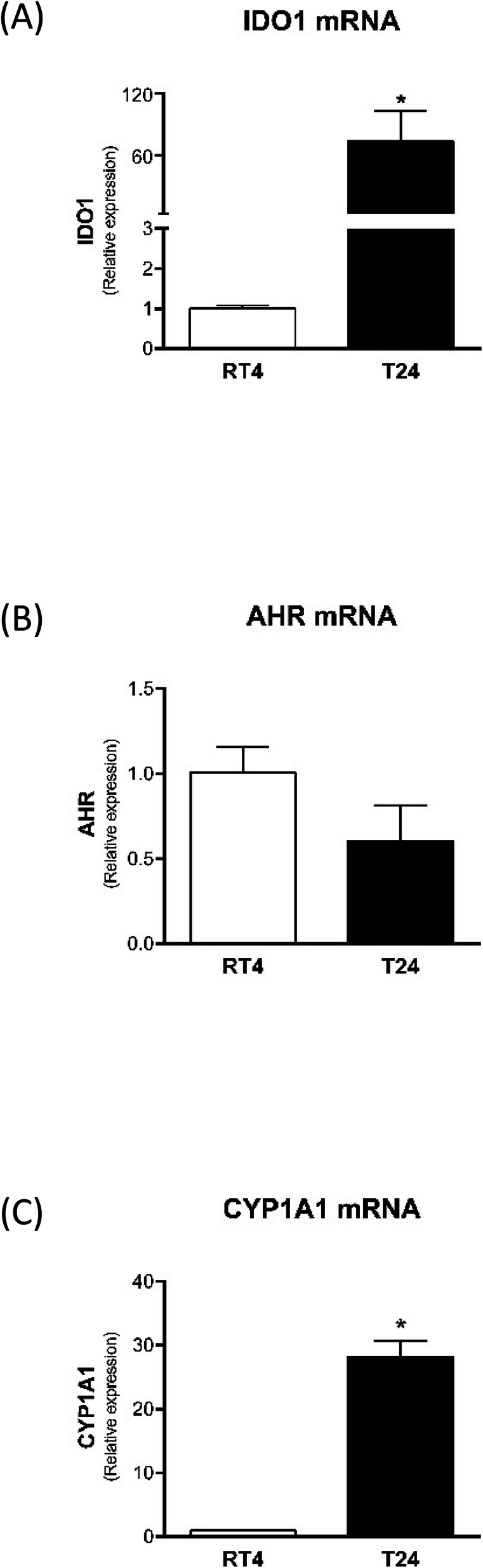
Expression of IDO1 (**a**), AHR (**b**), and CYP1A1 (**c**) evaluated by real-time PCR in grade 1 (RT4) and grade 3 (T24) BC cells. * *p* < 0.05 vs. RT4

Regarding cell culture experiments, IDO1 was constitutively expressed in both RT4 cells and T24 cells. However, T24 cells expressed significantly more IDO1 than RT4 cells (Fig. 1a). AHR expression was also observed in both cell lines, but no difference was observed between them (Fig. 1b). CYP1A1 was significantly increased in T24 cells (Fig. 1c).

### Effect of IDO1 inhibitors on BC cells

**Fig. 2:**
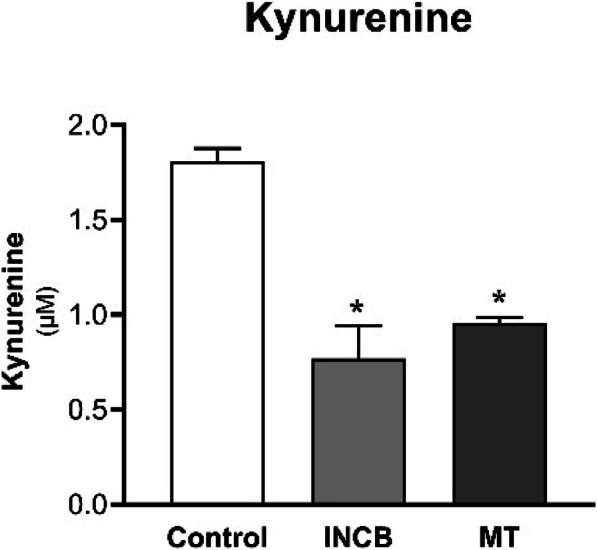
Kynurenine measurement in supernatant of T24 cells incubated with INCB240360 (INCB) or MT for 48 h. Kynurenine was measured using HPLC. * *p* < 0.05 vs. Control

Kynurenine was not detected in the supernatant of RT4 cells due to its low production. Therefore, it was impossible to evaluate the effect of the inhibitors INCB024360 and MT on kynurenine production. In contrast, a quantifiable amount of kynurenine was found in the supernatant of T24 cells, and the incubation with both inhibitors significantly decreased the kynurenine production (Fig. 2).

**Fig. 3:**
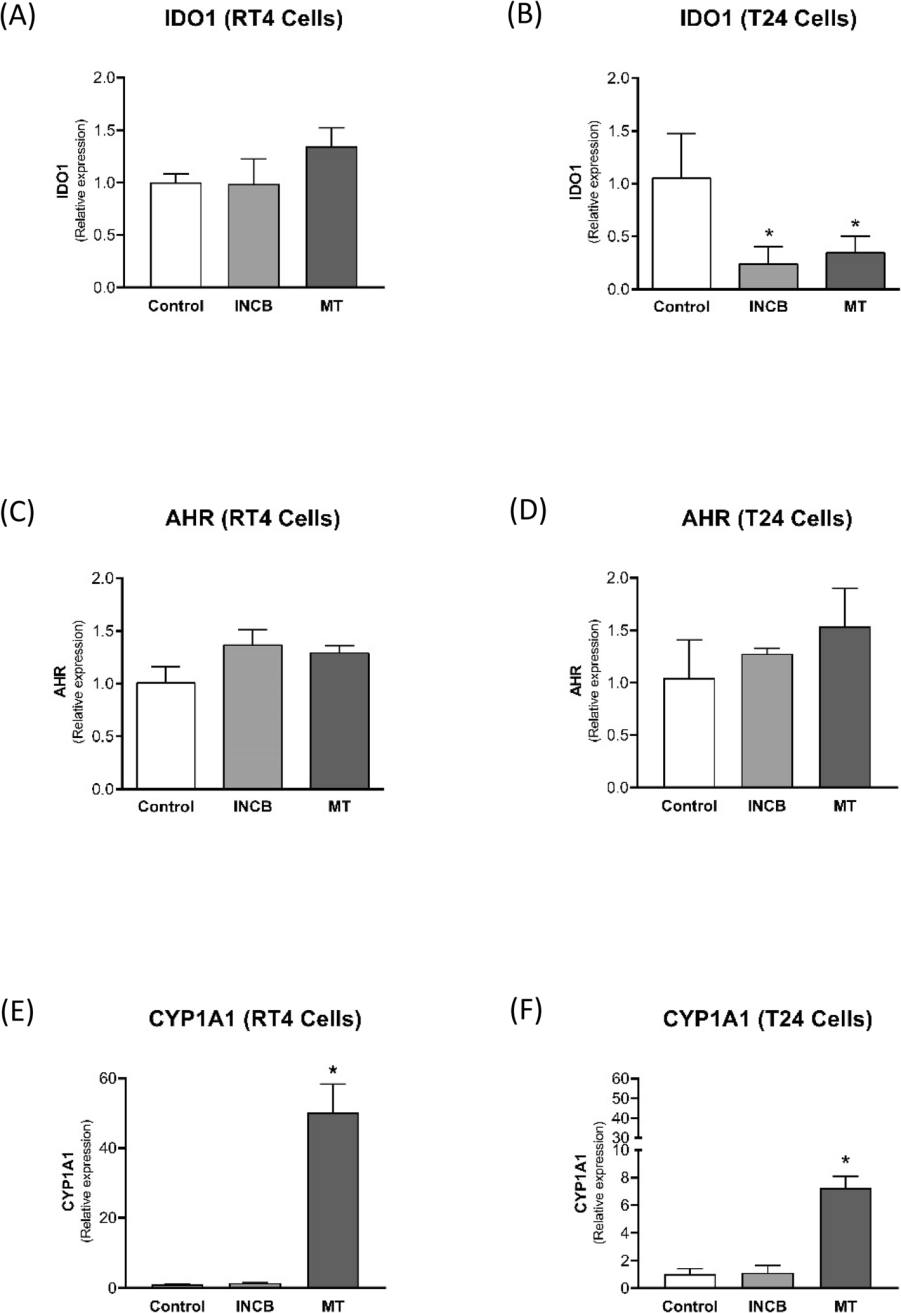
Effect of IDO inhibitors INCB240360 (INCB) and MT on the expression of IDO1 (**a and b**), AHR (**c and d**), and CYP1A1 (**e and f**) in RT4 and T24 BC cells. The cells were treated with IDO inhibitors for 48 h. The gene expression was analyzed by real-time PCR. * *p* < 0.05 vs. Control

The IDO1 inhibitors did not affect IDO1 expression in RT4 (Fig. 3a), however, they significantly reduced IDO1 expression in T24 cells (Fig. 3b). Regarding AHR expression, IDO1 inhibitors increased AHR expression in RT4 cells, having no effect on T24 cells (Fig. 3c and d). Finally, the expression of CYP1A1 was not affected by INCB024360 in both cell types; however, MT significantly increased CYP1A1 expression in RT4 and T24 cells (Fig. 3e and f).

## Discussion

AHR has been linked not only to the tumorigenesis initiation, but also to the promotion, progression, and metastasis [16]. In BC, AHR has been pointed as a potential inductor of carcinogenesis [17], but its correlation with tumor progression had not yet been demonstrated. AHR/ARNT complex activates several genes that encode cytochromes. Therefore, cytochromes expression has been used to identify AHR activation [18]. Here, using GEO datasets, we showed that the expression of CYP1A1, CYP1A2, and CYP1B1 was associated with grade, stage, and tumor progression in BC patients (Table 1).

The involvement of AHR in the carcinogenesis is reinforced by the action of 2,3,7, 8-tetrachlorodibenzo-p-dioxin, a compound that exacerbates the induction of cytochrome P450 enzymes via AHR ligation. Long-term treatment with this compound leads to the development of tumors of the oral cavity, liver, lung, thyroid, skin, bladder, and other sites [19]. Endogenous compounds may constantly activate AHR to sustain tumor promotion and progression. The inflammatory microenvironment developed into the tumors may set up autocrine/paracrine pathways, leading to sustained AHR activation. On the other hand, AHR knockout mice developed significantly more hepatic tumors when compared to wild type mice [20]. This phenomenon was also similar in prostate cancer mouse model [21]. Nevertheless, these studies suggest that AHR expression attenuates carcinogenesis while AHR activation enhances this process [16]. In our study, the AHR expression was not different between the BC patients. The observed difference was only in the expression of cytochrome P450 enzymes, which indicates higher AHR activation in advanced tumors. These findings are similar to other tumors. In terms of tumor progression and metastasis, AHR activation enhanced gastric cancer cell invasiveness, in part by induction of MMP-9 expression [22]. More evidences have also raised from non-neoplastic cells. AHR modulates adhesion proteins in smooth muscle cells to facilitate cell invasion [23]. In addition, AHR activation modulates transcription factors responsible for epithelial-mesenchymal transition, increasing the invasion ability of keratinocytes [24].

A strong relationship between AHR and IDO1 has emerged. Many catabolites formed by IDO1 activity are ligands of AHR. For example, the interaction of kynurenine with AHR modulates the generation of regulatory T cells [25]. Therefore, the immune escape mechanisms promoted by IDO1 include the activation of AHR. Additionally, AHR signaling induces dendritic cells to express IDO1, suggesting a feedback mechanism between AHR and IDO1 triggered by the tumoral inflammatory conditions [26]. The expression of AHR and IDO1 is not exclusive of the immune cells, in other words neoplastic cells also coexpress these molecules [27]. Here, we showed that either RT4 or T24 cells express AHR constitutively, however, CYP1A1 expression was significantly higher in T24 cells. Considering that RT4 cells are representative of grade 1 BC and T24 cells of grade 3 BC, the in vitro data are in accordance to the GEO datasets findings, where cytochromes were more expressed in grade 3 BC than in grade 1 BC (Table 1). This increase in the CYP1A1 expression found in T24 cells could be explained by the elevated expression of IDO1 in the same cells (more than 50X higher than in RT4 cells), an effect that would be intermediated by the high production of kynurenine. In fact, kynurenine was detectable only in T24 cells by HPLC, reinforcing the hypothesis that kynurenine would be activating AHR and consequently leading to the increased CYP1A1 expression in T24 cells. However, the use of the IDO1 inhibitor INCB024360 did not inhibit CYP1A1 expression in both RT4 and T24 cells. It is possible that there are other AHR ligands sustaining its activation in these cells independently of kynurenine. Another hypothesis is that the reduction of kynurenine reached with the IDO1 inhibitors was not sufficient to diminish CYP1A1 expression. Further experiments are needed to clarify this point. In the case of MT, a significant increase in CYP1A1 expression was observed in both cell lines. This finding corroborates the Lewis’ study in which MT increased CYP1A1 in mesenchymal cells [11]. The ligation between AHR and MT is chemically possible because MT contains an aromatic ring like other tryptophan derivatives. However, there is no study demonstrating this hypothesis. Further studies are needed to describe the exact MT moiety responsible for the AHR activation. Furthermore, it is important to know whether this effect occurs in other cell types. In BC this compound may not be recommended.

Our study is limited in terms of molecular mechanisms. Herein, we have no demonstrating the mechanisms by which AHR activation influences BC progression neither even how MT could impact this phenomenon through AHR activation. Further studies are necessary to clarify these mechanisms.

## Conclusion

In conclusion, AHR activation is associated with grade, stage, and progression of BC. Because part of the AHR activation is maintained by IDO1 activity via the formation of kynurenine and its downstream catabolites, it is reasonable to suggest the inhibition of IDO1 activity in BC disease. However, the inhibition with MT may not be recommended due to its effect in inducing AHR activation. The selection of IDO1 inhibitors for the treatment of BC must be careful, and INCB024360 appears to be a promising alternative.

## Data Availability

The microarray datasets were obtained from the GSE13507 series, at the Gene Expression Omnibus (GEO) (www.ncbi.nlm.nih.gov/geo) repositories, with relative expression of AHR, CYP1A1, CYP1A2, and CYP1B1 determined with the GEO in-built tool GEO2R. Patient data of tumor grade, stage and progression for the GSE13507 datasets was obtained from the studies of Lee and coworkers [13] and Kim and coworkers [14].

## Abbreviations

BC: Bladder cancer
AHR: Aryl hydrocarbon receptor
CYP: Cytochrome P450
IDO1: Indoleamine 2, 3-dioxygenase-1
MT: 1-methyl-D-tryptophan
